# Additively manufactured flexible on-package phased array antennas for 5G/mmWave wearable and conformal digital twin and massive MIMO applications

**DOI:** 10.1038/s41598-023-39476-w

**Published:** 2023-08-02

**Authors:** Kexin Hu, Yi Zhou, Suresh K. Sitaraman, Manos M. Tentzeris

**Affiliations:** 1 Georgia Institute of Technology, School of Electrical and Computer Engineering, Atlanta, 30308 USA; 2 Georgia Institute of Technology, George W. Woodruff School of Mechanical Engineering, Atlanta, 30308 USA

**Keywords:** Electrical and electronic engineering, Electronic devices

## Abstract

This paper thoroughly investigates material characterization, reliability evaluation, fabrication, and assembly processes of additively manufactured flexible packaging and reconfigurable on-package antenna arrays for next-generation 5G/mmWave wearable and conformal applications. The objective is to bridge the technology gap in current Flexible Hybrid Electronics (FHE) designs at mmWave frequencies and address the challenges of establishing future design standards for additively manufactured flexible packages and System-on-Package (SoP) integrated modules. Multiple 3D printed flexible materials have been characterized for their electrical and mechanical properties over the 5G/mmW frequency band (26–40 GHz), and the inkjet printed interconnects on 3D printed Polypropylene (PP) substrates demonstrated excellent electrical and mechanical performance during a 10,000-time cyclic bending test over typical wearable flexible radii down to 1 inch. A proof-of-concept flexible on-package phased array with an integrated microfluidic cooling channel on 3D printed substrates was fabricated and measured, demonstrating $\pm 37^{\circ }$ beam steering capability with efficient cooling. The proposed reconfigurable design and low-temperature fabrication approach using additive manufacturing can be widely applied to next-generation highly-complex on-demand FHE, flexible multi-chip-module integration, and on-package phased-array modules for 5G/mmWave wearable and conformal smart skin, digital twin and massive MIMO applications.

## Introduction

Next-generation data-driven Internet of Things (IoT) systems, large-scale smart cities, and wearable electronics for health monitoring are promised by the recent development of 5G/mmWave technologies with broadband operation and ultra-high data rates^1^. This has created an increasing demand for future System-on-Package designs with complex functionalities in compact modules, which require real-time response, deployable packaging, and highly-integrated modules. These designs are essential for digital twins, structural health monitoring for megastructures, robotic applications with stringent (10,000+) bending requirements such as those for Industry 4.0 applications and conformal intelligent metasurfaces. Flexible hybrid electronics utilizing flexible materials and rigid ICs are widely adopted in wearable designs due to their flexibility, but most of the FHE-based electronic systems are operating in low-frequency (e.g., MHz) because it is challenging to satisfy all the requirements for mmWave operation, including reliable performance within broad operational bandwidth, low parasitic losses from interconnects, and high gain phased array with beamforming abilities to overcome high path loss^2^. Additionally, these systems should be conformal in form factor with resilient and adaptable performance to bending and on-body effects from both RF and mechanical perspectives^3^, such as massively scalable MIMO enabling the implementation of antenna arrays with thousands of elements on virtually every conformal surface. However, most papers listing massive MIMO in the past lack practical deployment approaches.

**Figure 1:**
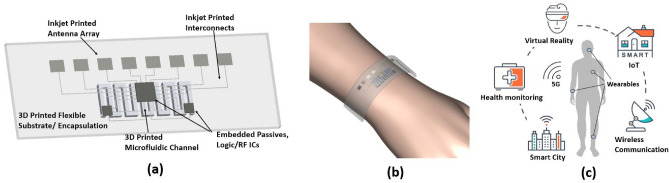
(**a**) Proof-of-concept demonstration of flexible packaging and wearable SoP modules utilizing additive manufacturing. (**b**) An example of the flexible SoP module used as a wristband. (**c**) Future applications of 5G wearable devices.

The solution proposed in this work is to utilize additive manufacturing (AM) techniques, including 3D printing and inkjet printing, to create on-demand customizable mmWave flexible packaging and reconfigurable on-package phased arrays for 5G wearable applications. A general demonstration of the proposed idea is shown in Fig. 1a. This type of flexible SoP can be applied to various wearable and conformal applications as shown in Fig. 1b,c. Conventional FHE designs utilize inkjet printing and commercially available flexible substrates, such as PET and Kapton^4–6^, but the main limitation of these materials is that they cannot support customized packaging designs with complicated structures. On the other hand, the combination of 3D printing and inkjet printing has enabled the production of highly customized and intricate electronic devices with lower costs and less waste of materials. Previous research has extensively studied the electrical and mechanical properties of 3D printed and inkjet printed materials^7,8^, but very little attention has been given to the material characterization over the mmWave band. Although some past designs have demonstrated the 3D printed antennas, most of them focused on 3D printed rigid materials, such as horn antennas^9^ and dielectric lenses^10^; only a few reported flexible antennas with 3D printed substrates, including patch antennas below 5 GHz^11^ and dielectric reflectarrays at 28 GHz^12^. Recently, the first flexible mm-wave on-package antenna with an embedded energy harvester has been reported^13^, utilizing SLA printing and inkjet printing. The flexible module achieved 0.9 V output power at a 20 cm range with 59 dBm EIRP. Nonetheless, various challenges persist in implementing additively manufactured multi-chip modules on 3D printed flexible substrates. For example, conductive epoxy was used to attach a diode onto flexible substrates in^13^ because the 3D printed polymer substrates are sensitive to heat and cannot support thermal processing like soldering, and this poses a challenge for the accurate attachment of mmWave ICs due to their smaller pad and pin size. Heat dissipation of the IC may also become a problem with many flexible polymer substrates because their thermal conductivity is not as good as that of traditional rigid copper substrates like Rogers^14^. Another gap in past research is the lack of reliability evaluation of these flexible packages. Although all previous AM module applications refer to single bending-if any-, this is the first paper proposing 10,000+ bending reliable performance operability for practical bending radii for wearable and conformal 5G+ applications. The utilization of different additive manufacturing technologies has made FHE designs more accessible to in-house fabrication, but without generalized standards, it is difficult to evaluate the adaptability of the process and the quality of the designs.

In this work, the aforementioned challenges and technology gaps have been successfully addressed. Different flexible materials from both Fused Deposition Modeling(FDM) printing and Stereolithography (SLA) printing were characterized for their electrical and mechanical properties over 26–40 GHz, covering the 5G mmWave frequency band. The fabrication process of inkjet printing onto 3D printed substrates has been optimized, and the fabricated microstrip line interconnect samples on 3D printed low-loss Polypropylene substrates have demonstrated less than 0.1 dB/mm insertion loss over 24–40 GHz, even under reliability evaluations through 10,000 times cyclic bending tests over a 1-inch bending radius. Finally, an on-package phased array with an integrated microfluidic cooling channel was designed, fabricated, and assembled through a low-temperature process, as a proof-of-concept demonstration for the proposed packaging concept. The implementation of a flexible phased array in this design allows additional reconfigurability that can be attached to any kind of surface while maintaining the antenna performance. The comprehensive investigation presented in this paper, including material characterization, fabrication process, reliability evaluation, and SoP implementation of additively manufactured 5G mmWave flexible packages, can provide guidelines for future FHE designs, and build the foundation of next-generation wearable technologies for various applications.

## Additively manufactured material characterizations

### Electrical properties of 3D printed flexible materials

Accurately characterizing the electrical properties of 3D printed flexible materials is the first step to designing flexible hybrid electronics systems. Even materials of the same type from different manufacturers can exhibit variations in their dielectric properties due to differences in fabrication methods and material composition^15^. Thus, it is necessary to create a comprehensive library of common 3D printed flexible materials for different applications. Insertion loss is one of the key performance parameters to evaluate substrate materials at mmWave frequencies because generating and maintaining signal power at mmWave frequencies is significantly more challenging than at lower frequencies, and minimizing insertion loss is crucial for ensuring high-quality and reliable signal transmission at mmWave frequencies^16^. The material properties of substrates, such as electric permittivity and surface roughness, can significantly impact the insertion loss. In particular, the real part of the permittivity is linked to dipole relaxation and lattice vibrations in the mm-wave range. The imaginary part of permittivity, known as loss tangent, corresponds to the ion friction effect that influences the loss properties of materials^17^. The typical value for loss tangent of a commercial substrate for mmWave applications is smaller than 0.01.

**Figure 2:**
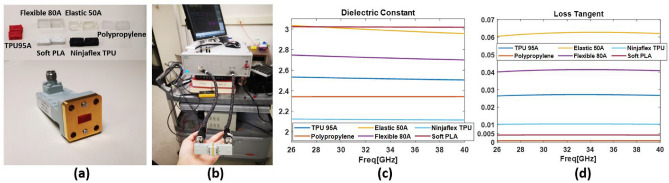
Dielectric properties of 3D printed flexible materials over 26–40 GHz. (**a**) WR28 waveguide with the sample holder and 3D printed material samples. (**b**) Measurement setup. (**c**) Extracted dielectric constant. (**d**) Extracted loss tangent.

In this study, 6 different types of 3D printed flexible materials were considered^18^: Flexible 80A and Elastic 50A from Formlabs 3 (SLA printer); TPU 95A, Polypropylene, Soft PLA, and Ninjaflex TPU from Ultimaker S3 (FDM printer). They represent common types of commercially available 3D printed flexible materials. Transmission/reflection method^19^ was used to perform wideband characterization of the selected dielectric materials from 26–40 GHz. Samples of 3D printed materials were measured using WR28 waveguides and a Shockline 552B Vector Network Analyzer. To hold the samples, a waveguide sample holder of 2.99 mm thickness was used, and all materials under test were 3D printed as small cubes of size $3.556~\textrm{mm} \times 7.112~\textrm{mm} \times 2.99~\textrm{mm}$. The measurement results were processed in Matlab, combined with the measured S parameters for an empty sample holder to derive the dielectric constant and loss tangent for each material using the Nicolson–Ross–Weir (NRW)^20^ method, which is the most straightforward algorithm in extracting dielectric properties by processing S parameter results. At least three samples were measured for each material, and the extracted properties were calculated in average values. Figure 2 displays the waveguide sample holder, 3D printed samples of various materials, and the measurement setup. The results presented in Fig. 2 and Table 1 show that the dielectric properties of these materials have minimal variations in the 26–40 GHz frequency band. The materials with larger dielectric constant and loss tangent are Flexible 80A and Elastic 50A from Formlabs, while Polypropylene (PP) printed from Ultimaker features a very low loss tangent of 0.001, making it preferable for mmW flexible modules.

Surface roughness also needs to be taken into consideration when utilizing 3D printed materials for FHE designs. Since inkjet printing is commonly used as one of the metallization methods, the poor surface roughness of the substrates will lead to low print quality and high conductor loss^21^. FDM printers typically generate surfaces with uneven patterns, thus, accurate surface morphology characterization is necessary to evaluate the properties of different materials. It should be noted that surface roughness also depends on the printing setup, and the resulting surface quality can differ depending on the printing pattern^22^. All the FDM printed substrates used in this paper were printed with the typical zig-zag pattern. The highest surface roughness is measured along the perpendicular direction to the hatching pattern, and the resulting roughness values (Rq) are summarized in Table 1. Compared to FDM printed materials, those printed using stereolithography (SLA) tend to exhibit smoother surfaces. Among the materials tested, Polypropylene (PP) demonstrates the lowest surface roughness and therefore shows the potential as a substrate for inkjet printing.

**Table 1:** Electrical and mechanical properties of 3D printed flexible materials over 26–40 GHz.

Material	3D printing technology	Dielectric constant	Loss tangent	Surface roughness Rq (um)	Elastic modulus (MPa)	Poisson’s ratio
Flexible 80A	SLA	2.73	0.041	0.641	7.69	0.13
Elastic 50A	SLA	3	0.062	0.902	2.89	0.23
TPU 95A	FDM	2.52	0.027	5.68	32.4	0.36
Polypropylene	FDM	2.34	0.001	0.063	218.15	0.3
Soft PLA	FDM	3.02	0.004	0.312	155.17	0.3
NinjaFlex TPU	FDM	2.12	0.01	1.74	11.42	0.36

### Mechanical properties of 3D printed flexible materials

The mechanical properties of 3D printed flexible materials mainly influence the range of applications of FHE designs. For instance, in wearable devices, the substrate material needs to be flexible and stretchable to conform to the shape of the human body and allow for comfortable and continuous wear. It needs to be durable enough to withstand repeated bending, stretching, and twisting^23^. The mechanical properties of the substrate material also affect the performance and reliability of the functional materials and components printed on top of it, such as conductive traces, sensors, and batteries. For example, if the substrate material is too rigid, it can cause cracking or delamination of the printed components, leading to the failure of the device.

**Figure 3:**
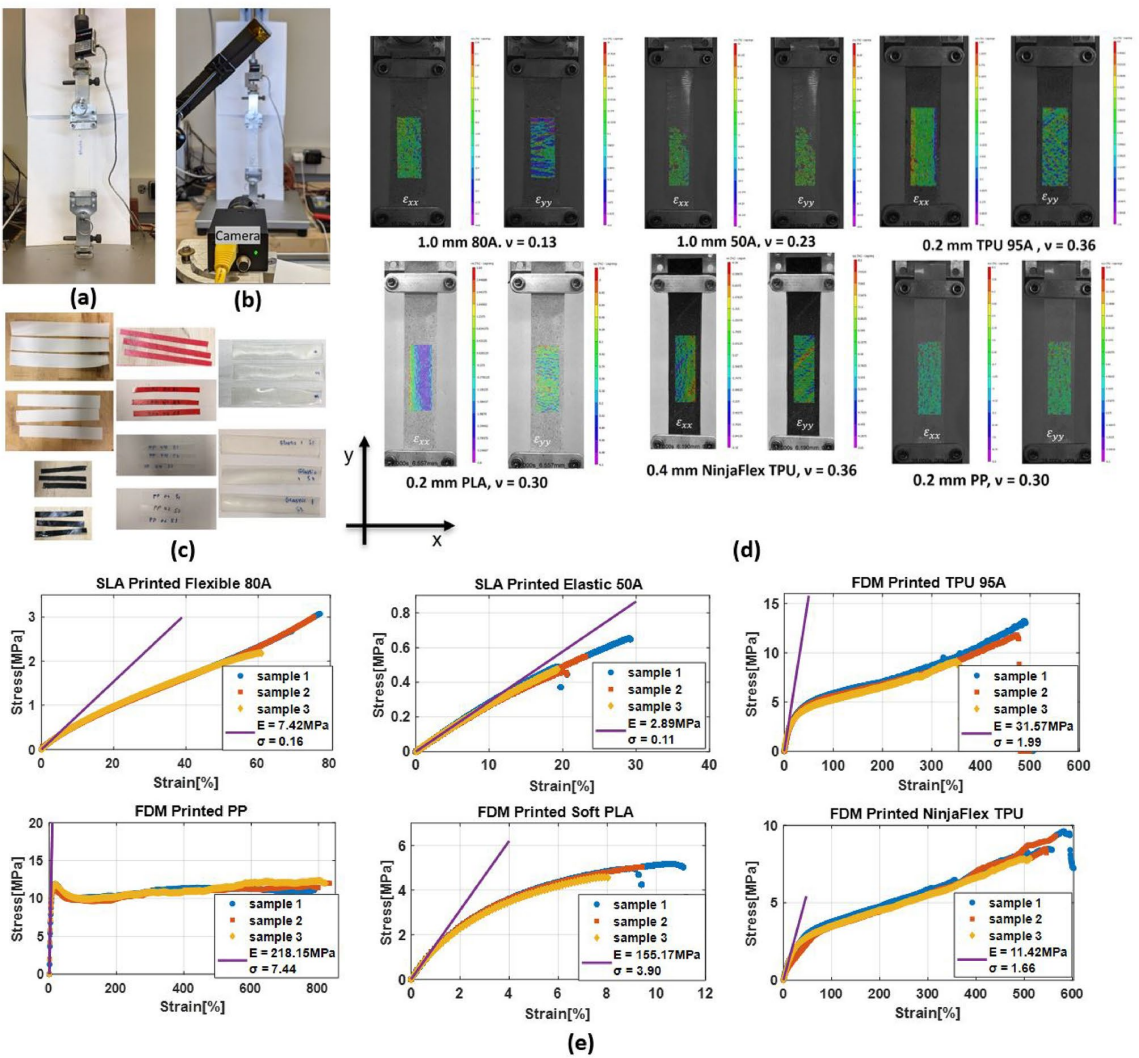
Mechanical properties of 3D printed flexible materials (**a**) Uniaxial test setup by universal testing machine loading with a standard size sample. (**b**) DIC setup with a camera. (**c**) 3D printed materials samples for stretching tests. (**d**) DIC processed transverse and longitudinal strain distribution of 3D printed materials. (**e**) Stress–strain curves of the tested 3D printed materials.

This study utilized a universal testing machine (Fig. 3a) to conduct uniaxial stretching tests on six different 3D printed materials^18^, resulting in stress–strain curves. The curves were used to measure the elastic modulus, percent elongation at break, and tensile strength (nominal) of the materials. Additionally, a 2D digital imaging correlation (DIC) system (Fig. 3b) was employed to obtain the Poisson’s ratios of the materials. Standard sample sizes were used to characterize all six materials, except for 80A, 50A, and Soft PLA, which required a smaller size to obtain the complete stress–strain curve, as shown in Fig. 3c. Three samples were tested for each material, with an additional sample for DIC. The stress–strain curves were based on actual measurements to consider variations in the printed thicknesses. The findings showed that PP had the lowest surface roughness, an elastic modulus of 230 MPa, and a Poisson’s ratio of 0.40. Unlike the other materials, PP could withstand up to 800 strains without breaking, making it suitable for wearable SoP structures. The mechanical properties of all six materials are shown in Fig. 3 and summarized in Table 1.

### Ink characterization

Inkjet printing technology has gained significant attention in the field of flexible and stretchable electronics due to its many advantages. As a low-cost and versatile additive technique, it enables the deposition of precise patterns with high resolution and accuracy, making it ideal for printing complex patterns and fabricating multi-layered structures^24^. It supports the use of various types of inks, including conductive, dielectric, and biological inks, as well as a wide range of substrates, including flexible, stretchable materials and 3D printed structures. Furthermore, inkjet printing is also a highly-scalable process, allowing for the rapid and cost-effective production of large-scale and high-volume devices^2^.

**Figure 4:**
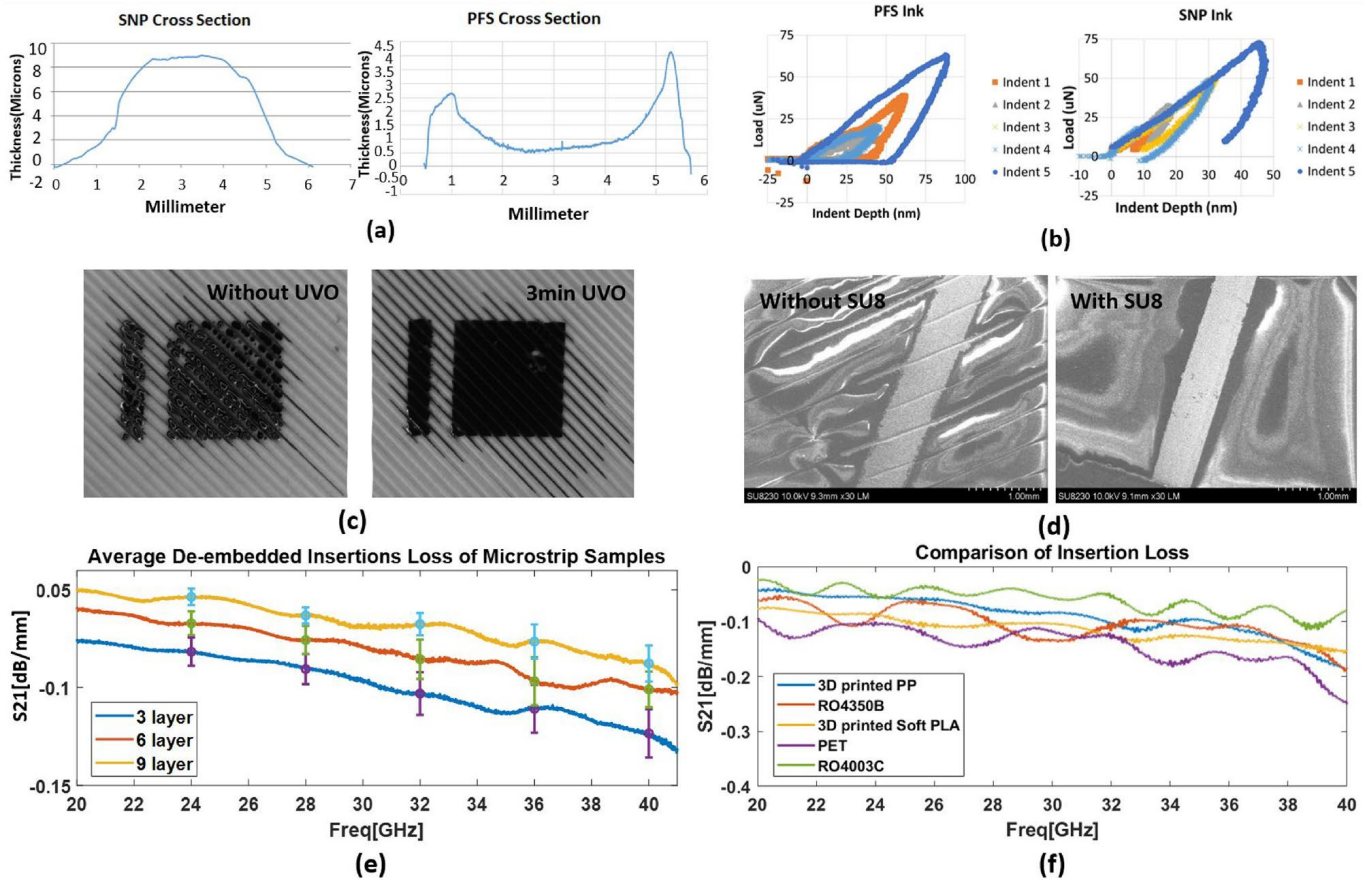
(**a**) Cross-section measurements of SNP ink and PFS ink. (**b**) Nanoindentation of SNP ink and PFS ink. (**c**) Comparison of inkjet printed SNP ink on PP substrates without and with UVO surface treatment. (**d**) Comparison of inkjet printed SNP ink with and without SU8 surface roughness reduction layers on PP substrates. (**e**) Measured de-embedded insertion loss of microstrip line samples with different metallization thicknesses. (**f**) Comparison of Insertion Loss of inkjet printed microstrip line samples on different substrates.

In this study, an inkjet printer, Fuji Dimatix DMP-2850, and 10 pL cartridges were used to print conductive inks with a 20 um drop spacing for 8 layers onto a PET substrate for ink characterization^18^. The conductivity and elastic modulus of two types of ink were measured: Silver nano particle (SNP) ink EMD5730^25^ from Suntronic and Particle free silver (PFS) ink from Electroninks, which are commonly used conductive inks for metallization of different flexible substrates. The SNP ink was sintered at 130 $^\circ$C for 1 h, while the PFS ink was sintered at 160 $^\circ$C for 30 min in the oven. The measured cross sections are shown in Fig. 4a. The PFS ink exhibited a strong coffee ring effect, which resulted in uneven drying and accumulation of the ink primarily along the perimeter of the printed pattern. The calculated conductivity is $1.73 \times 10^7 \mathrm{S/m}$ for PFS ink and $1.68 \times 10^6 \mathrm{S/m}$ for SNP ink. To be an effective conductor at mmWave frequencies, conductive materials must not only have high conductivity but also achieve a thickness of at least 3 skin depths^26^. The skin depth is the distance below the surface of a conductor where the current density has diminished to 1/e of its value at the surface. It is inversely proportional to the conductivity of the silver ink at certain frequency. At 30 GHz, SNP ink needs at least 2.205 um, while PFS ink needs to have at least 1.35 um. Considering the coffee ring effect of PFS ink after sintering, it would be difficult to uniformly satisfy the skin depth requirements in the cross-section, meaning the effective resistance will significantly increase, thus bringing in additional conductor loss. Additionally, the required sintering temperature of the selected PFS ink is much higher than that of the SNP ink, which could pose a challenge to many 3D printed polymer substrates that are highly sensitive to thermal treatment. A potential solution to this challenge is to use UV sintering instead of heat sintering to avoid any potential thermal damage to the substrate.

The nanoindentation technique was employed to obtain the elastic moduli of two inks^18^. Specifically, a 1 cm $\times$ 1 cm square was printed using each ink, and the Hysitron Triboindenter was used to perform the nanoindentation. To obtain accurate material properties, it was crucial to ensure that the loading portion of the curves followed the same trend as the unloading portion and that the indentation depth was significantly smaller than the thickness of the ink layer. Nine indents were performed on each of the ink, with the load linearly increasing from 25 to 100 uN. Figure 4b displays the valid indentation data obtained from the two inks, excluding those that do not follow the loading trend due to the voids of the printed ink. The PFS ink demonstrated an average elastic modulus value of 2.08 GPa, while the SNP ink demonstrated an average elastic modulus value of 7.72 GPa.

## Evaluation of inkjet printed microstrip lines and interconnects

### Inkjet printing onto 3D printed substrates

Evaluation of the fabrication process is essential for the successful design and manufacturing of FHE devices. It is necessary to optimize the inkjet printing process onto 3D printed substrates, to understand the capabilities and limitations, and improve the reliability and performance of the final prototype. To evaluate the inkjet printability of conductive ink on different materials, SNP ink was utilized in this study. The printing quality of silver ink on 3D printed materials is mainly influenced by surface wetting and surface roughness. Surface wetting can be enhanced by employing UV Ozone treatment, which is an efficient and simple technique for increasing the hydrophilicity of plastic surfaces. The duration of UVO treatment may differ for various substrates. For instance, in the case of PP substrate, it has been found that 3 minutes of cleaning can lead to the best printing quality for SNP ink^27^, as shown in Fig. 4c. Surface roughness, on the other hand, is caused by the printing techniques and features, as discussed in earlier sections. A feasible way to reduce surface roughness is to sand the surface of the rough materials utilizing a hand sander. For example, SNP ink adheres very well to SLA printed materials such as Flexible 80A and Elastic 50A after sanding. However, for FDM printed materials, the uneven pattern caused by printing cannot be eliminated by sanding. Therefore, a buffer layer is required between the silver ink and substrates. SU-8 can be used for this purpose^27^, which is an epoxy-based photoresist that can be cured by a UV crosslinker, with a dielectric constant of 3.2 and a loss tangent of 0.04 at 24.5 GHz^28^. Figure 4d compares the surface morphology of inkjet printed silver trace on PP substrate with and without SU8 buffer layers. It is evident that with the SU8 coating, the uneven pattern disappears, and the silver trace becomes much smoother. This significantly enhances the print quality and minimizes the chance of cracking during bending.

To validate the electrical properties of 3D printed PP substrates and confirm the feasibility of the fabrication process, microstrip line samples were fabricated on 3D printed PP substrates using the inkjet printing process as mentioned above. Different metallization thicknesses of 3, 6, and 9 layers of silver ink were printed, and the average de-embedded insertion loss of different prototypes was shown in Fig. 4e. The error bars indicate the standard deviation at significant frequencies for 5G NR bands: 24 GHz, 28 GHz, 32 GHz, 36 GHz, and 40 GHz. The results revealed that the 9-layer samples had the lowest loss, and the insertion loss of all the samples with different ink thicknesses was below 0.15 dB/mm, which is consistent with the difference in conductivity and low loss tangent feature of PP substrate. The standard deviations in insertion loss increased as the frequency increased because higher frequencies are more sensitive to surface imperfections. However, the standard deviation values of all three sample thicknesses were similar and relatively small, indicating good repeatability in the sample fabrication. As a benchmark, microstrip line samples were also fabricated on 3D printed soft PLA, PET, RO4350B, and RO4003C substrates. The dielectric properties, substrate thickness for each material, as well as the corresponding line width to achieve impedance matching at 50 Ohm, are recorded in Table 2. All samples were of uniform length (3 cm) and measured using the same VNA. The effect of end launch connectors was removed from the de-embedding process. The de-embedded insertion loss was recorded and plotted in Fig. 4f and summarized in Table 2. The average S21 was calculated as the mean between the maximum and minimum insertion loss over 20–40 GHz. The results demonstrated that the S21 measurement values were consistent with the dielectric properties of the different substrates. Specifically, the PP substrate exhibited low insertion loss, less than 0.1 dB/mm up to 40 GHz, comparable to commercially available RF substrates such as RO4003C and RO4350B.

**Table 2:** Inkjet printed microstrip line samples on different substrates.

Material	Dielectric constant	Loss tangent	Substrate thickness	Trace width	S21 (dB/mm)
Polypropylene	2.34	0.001	0.2	0.55	0.07
Soft PLA	3.01	0.004	0.2	0.5	0.1
RO4350B	3.66	0.0037	0.17	0.35	0.1
RO4003C	3.38	0.0027	0.203	0.47	0.075
PET	2.9	0.008	0.12	0.33	0.15

### Reliability of inkjet printed microstrip lines and interconnects

**Figure 5:**
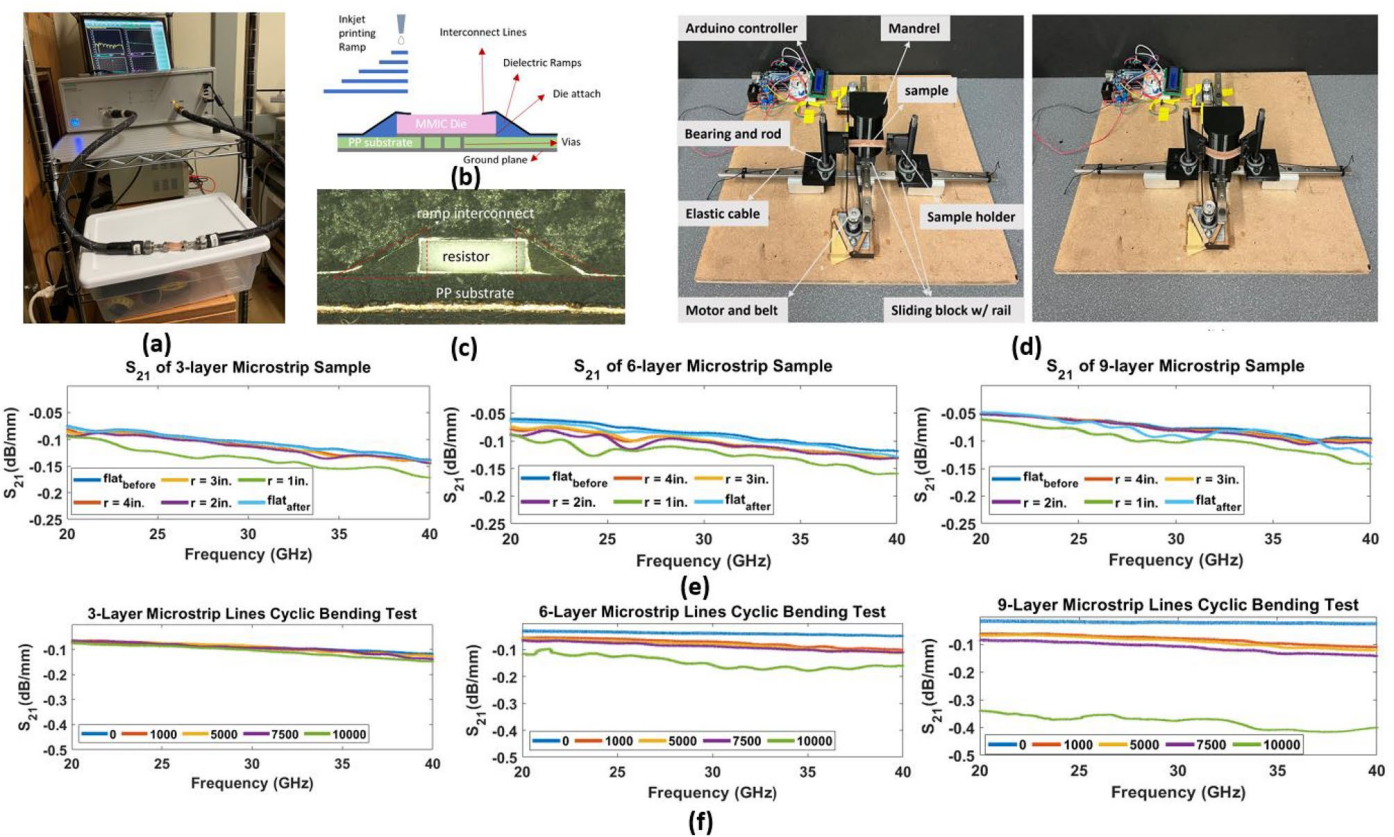
(**a**) RF measurement setup for microstrip line samples. (**b**) Cross-section schematic of inkjet printed ramp interconnects for MMIC die on a 3D printed Polypropylene substrate. (**c**) Cross-section view of the ramp interconnects structure for reliability tests. (**d**) Cyclic bending tests system. (**e**) Measurement results of monolithic bending tests for bending radii 1–4 inches. (**f**) Measurement results of cyclic bending tests up to 10,000 cycles.

To further investigate the reliability of microstrip lines with different metallization thicknesses (number of inkjet printed conductive layers) using the proposed fabrication method, monolithic bending tests and cyclic bending tests were conducted^18^. Four sizes of cylindrical polycarbonate mandrels (outer radius: 1–4 inches) were used to sequentially bend microstrip lines wrapped around them, with the ink layer facing outward. S21 measurements were taken before and after bending, with the smallest mandrel inducing the largest insertion loss, as shown in Fig. 5. The insertion loss increases as the microstrip line samples experience the tensile strain at bending compared to the flat condition. The 9-layer sample showed the lowest loss in both the flat and bent configurations. All samples maintained good transmission at bending radii greater than 1 in, with minimal damage to the ink structure.

An automated mandrel testing system was then designed for cyclic bending tests. The experiments involved taking S21 measurements before and after 1000, 5000, 7500, and 10,000 bending cycles for three sets of samples comprising 60 mm long microstrip lines with 3, 6, and 9 layers of printed silver ink. The results, presented in Fig. 5, indicate that the insertion loss of the microstrip lines increased with the number of bending cycles, attributed to fatigue failures in the printed ink structure. However, the extent of insertion loss changes varied based on metallization thickness. While the 9-layer samples displayed the best electrical performance before bending, they exhibited the worst reliability. In contrast, the 3-layer samples showed stable S21 during bending cycles but low conductivity. The 6-layer samples exhibited an insertion loss smaller than 0.1 dB/mm for all frequency bands, making them the optimal choice for inkjet printing silver traces to achieve a balance between good electrical and mechanical reliability for 5G and mm-wave flexible and wearable applications.

Similar cyclic bending tests were also performed on inkjet-printed ramp interconnects. As mentioned in previous sections, one of the key requirements of mmWave packaging is the realization of fully printed interconnects that feature ultra-low loss and “rugged” mechanical integrity, even for challenging bending radii. Compared to traditional interconnects such as wire and ribbon bonds that suffer from longer loop lengths and higher parasitics at mmWave frequencies, inkjet-printed ramp interconnects offer more robust, planar, and conformal structures with improved RF performance even in challenging configurations^2^. Previous studies have demonstrated the fabrication of ramp interconnects through inkjet printing, aerosol jet printing, and 3D printing, and this paper is the first testing of the reliability of this type of interconnect up to 10,000 cycles. Two ramp interconnects were fabricated using SU-8 layers on both sides of a zero-ohm resistor and connected to 1.5 cm long microstrip lines that lead to two pads for resistance measurement, as shown in Fig. 5b,c. The measurement results showed that the resistance between the two pads remained constant at 3.5 $\Omega$ before and after 10,000 bending cycles. SEM images also indicated that there was no damage to the interconnects during the bending^27^. These findings can guide the design and fabrication of reliable inkjet printed SNP-based electronic designs for wearable and conformal applications in 5G and B5G mmW frequencies.

## Flexible wearable 5G/mmW phased array module with integrated microfluidics

### Design and fabrication

**Figure 6:**
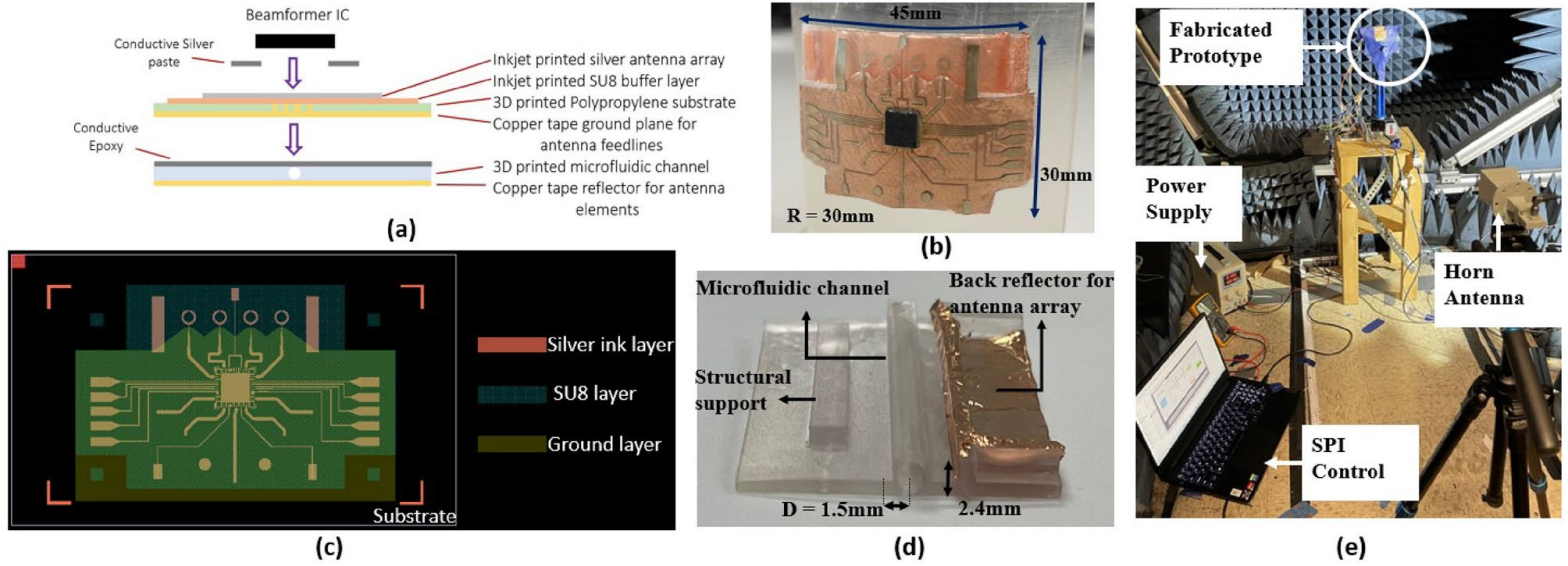
(**a**) Cross-sectional schematic of the fabrication process of the proof-on-concept module. (**b**) Top view of the flexible on-package phased antenna array bent over a curved surface with a 30 mm radius. (**c**) The layout of the phased array design. (**d**) Side view of the SLA printed microfluidic channel module. (**e**) Measurement setup.

To demonstrate the concept of smart packaging integration using the aforementioned additive manufacturing techniques, a 2.5D ultra-wideband (UWB) antenna array with embedded beamformer IC and the microfluidic cooling channel was fabricated on 3D printed Polypropylene substrate and Flexible 80A substrate^29^. Figure 6a–c show the overall design and cross-sectional schematic. The module is mainly composed of three parts: Anokiwave beamformer IC AWMF-0109^30^, inkjet printed monopole phased array on 3D printed PP substrate, and 3D printed microfluidic channel. The antenna element in this work is a circular monopole antenna with back reflectors, to achieve wideband performance covering the entire 5G mmW frequency band from 24 to 40 GHz. Conventional UWB antenna elements like horn antennas and Vivaldi antennas are relatively bulky^31^, which become challenging in compact integration. Monopole elements can be planar and miniaturized, but they demonstrate omnidirectional or bi-directional radiation patterns, which are not preferred in many applications, such as wearable devices requiring good flexibility and isolated radiation from the human body. The design proposed in this paper overcomes these limitations in terms of size and radiation pattern, by incorporating a back reflector and a side reflector with the circular loop monopole elements, enabling broadside radiation with enhanced beamforming and reduced side lobe levels. The design guidelines for circular ring monopole antennas can be found in past research^32^. The beamformer IC can integrate multiple channels, and each has independent phase and amplitude control in their transmit and receive paths, allowing the RF and microwave front end to electronically steer the antenna beams for an optimum transmission aiming pattern. To match the 50 Ohm impedance from each RF output port of the IC, the width of the antenna feedlines is designed to be 0.5 mm, which is calculated based on the relative permittivity (2.34) and the thickness (0.2 mm) of the 3D printed PP substrate^33^. Additionally, all the feedlines are designed with the same electrical length, allowing the beamformer IC to achieve beam steering by adjusting the phase shift between elements. The antenna array was inkjet printed onto the PP substrate using the same process as explained in the previous section.

Attaching the beamformer IC to the inkjet printed antenna array was the most challenging step during the assembly process due to the sensitivity of 3D printed polymer materials to thermal processes. Conductive epoxy is often employed in this regard, but its limitations include difficulty in effectively constraining the epoxy deposition within the pad area, which could cause a short between neighboring pins if there is a small misalignment of the IC. Additionally, the IC could detach easily when the epoxy dries up. An alternative solution is using Anisotropic Conductive Adhesive (ACA) material that is conductive only on the z-axis. The most recent research proved ACA materials to be very reliable and mechanically strong^34^. However, for the FDM printed substrate used in this paper, the uneven surface pattern makes it impossible for ACA to reach the uniform pressure condition to generate joints (small silver balls). On the other hand, traditional methods using conductive epoxy have shown higher conductivity but limited reliability^35^. Therefore, the method in this paper, using conductive epoxy for attachment and applying ACA to enhance mechanical strength, has achieved a balance between electrical connection and mechanical reliability.

To ensure accurate alignment of the IC pins, a stencil made from a transparent PET sheet was fabricated using a laser cutter. Conductive adhesive epoxy was applied to each pad of the inkjet printed IC footprint, and the IC was placed within the stencil. Uniform pressure was applied on top of the IC to ensure a solid electrical connection, and wires were attached to the DC pads for Serial Peripheral Interface (SPI) connection. Then an ACA material was used to seal the corners and edges of the IC to enhance mechanical connection. Vias were made through 0.2 mm diameter drilled holes filled with silver conductive paste, and the copper tape was used for ground plane metallization due to its quick fabrication and testing. The proposed approach can be readily applied to other flexible substrates, and it offers a practical solution for attaching components onto thermal-sensitive substrates.

As mentioned earlier, 3D printed polymer materials may suffer from poor thermal conductivity, which necessitates the integration of a microfluidic cooling channel for effective thermal management of the IC. In this work, the microfluidic channel was fabricated using Formlabs’ Flexible 80A material, printed as a cylindrical pipe with a diameter of 1.5 mm and walls that were 0.2 mm thick, and positioned underneath the IC’s center while attached below the antenna substrate layer^29^. A 1.5 mm diameter was chosen to allow for maximum water flow while maintaining 3D print tolerances and flexibility, as illustrated in Fig. 6d. Additionally, the module incorporated a copper tape back reflector for the antenna array and a 3D printed structural support. The smoothness of the channel walls was preserved during printing, and there was no need for internal support structures due to the use of SLA printing. Following printing, the module was washed in IPA for 20 min and cured under UV light for 10 min. The printed external support structures were then removed, and the antenna array was attached using conductive epoxy. Other thermal management options, such as air-cooled heat sinks, can also be 3D printed and integrated with the antenna module for different applications^36^.

**Figure 7:**
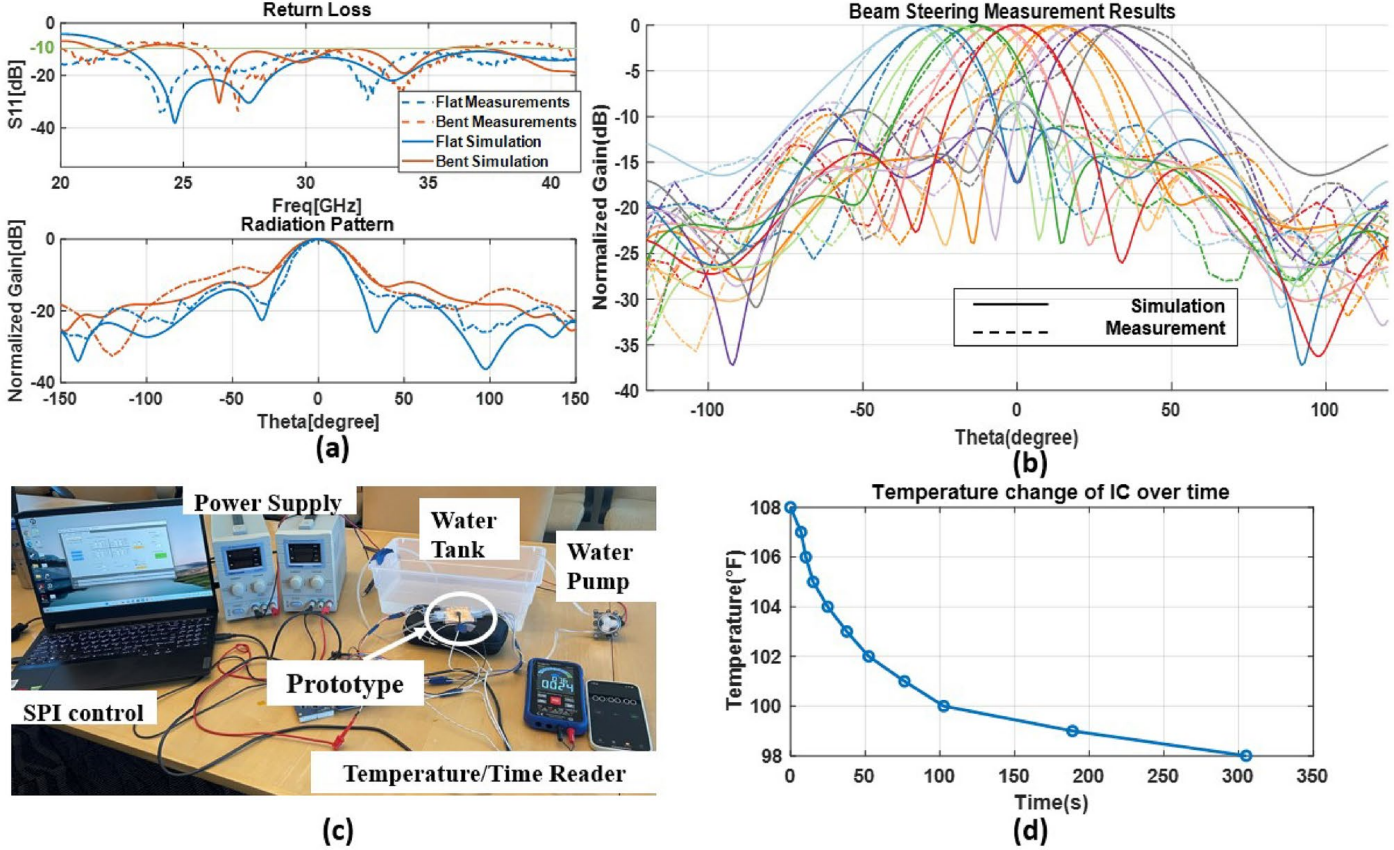
(**a**) Comparison of simulated and measured S11 values (top) and radiation pattern [H plane] (bottom) for the proof-of-concept prototype in flat and bent with a radius of 30 mm configurations. (**b**) Beam steering measurement results of the fabricated phased array module prototype with an integrated microfluidic channel. (**c**) Measurement setup for microfluidic cooling tests. (**d**) Cooling of the IC over time through the use of a microfluidic channel.

### Measurements and results

The measurements of the beamforming performance of the integrated phased array module were conducted in an anechoic chamber, as depicted in Fig. 6e. The DUT is in the center of the figure, attached to a rotating rod to cover a 360-degree radiation pattern. The S11 response and radiation pattern at 30 GHz were measured for both flat and bent conditions using a cylinder with a 30 mm radius to induce bending. A 30 mm bending radius was selected to represent the average size of a human wrist or arm to meet the flexibility requirements of most wearable applications.

The measurement results, as illustrated in Fig. 7a, closely agree with the simulation, and bending the antenna module was found to shift the operational frequencies while slightly increasing the beamwidth and side lobe level, due to the difference in impedance matching and element spacing. Nonetheless, the overall performance of the antenna under bending was acceptable. The beam steering capability of the inkjet printed phased array was evaluated under flat conditions, achieving $-37^{\circ }$ to 37$^{\circ }$ beam steering without grating lobes and a maximum side lobe level of $-8.45$ dB, as shown in Fig. 7b. The phase shift between the neighboring elements $\Delta \Phi$ is decided by the steering angle $\theta$ and the element spacing d of the phased array^37^:

1 $$\begin{aligned} \Delta \Phi = \frac{2\pi d\sin {\theta }}{\lambda } \end{aligned}$$

The measured maximum realized gain was 10.09 dBi, and the gain degradation was less than 4 dB between steering beams. The asymmetry and discrepancy of beamwidth compared with the simulation were attributed to the slight warpage and bending of the soft antenna substrate.

The microfluidic cooling performance was evaluated by pumping room temperature water (70 $^{\circ }$F) through the channel at a rate of 1.45 mL/s using a Yanmis 12 V peristaltic pump, and the temperature was measured using a Kaiweets HT 118A thermocouple^29^ (Fig. 7c). The initial measured temperature was 108 $^{\circ }$F when the IC consumed the maximum power. The steady-state temperature value was reached after around 5 min at 98 $^{\circ }$F, as shown in Fig. 7d. Assuming a thermal conductivity of 0.283 W/m*K for Flexible 80A and a rectangular cross-sectional area, the heat equation yielded a total heat dissipation of 1.052 W under maximum operating temperature conditions^38^.

## Discussion

Through material characterization, fabrication process, and SoP implementation, this paper presents the first and most thorough study covering all the important steps in 5G mmWave FHE designs utilizing inkjet printing and 3D printing. A novel fully additively manufactured 2.5D on-package reconfigurable antenna array with an integrated cooling solution was also reported. It overcomes the challenges in traditional designs in terms of size, flexibility, and IC assembly. Although additive manufacturing techniques used in this design have significantly improved customization and flexibility, and largely reduced fabrication cost and iteration time, the trade-off is the utilization of additional assembly steps for components on 3D printed substrates, compared with one-step soldering on traditional substrates. Nevertheless, the results and approaches reported in this paper provide valuable direction on design, fabrication, and integration for future flexible packages. Inkjet printing and 3D printing technologies offer significant potential for enhancing the performance and functionality of flexible electronic systems. The evaluation of electrical and mechanical properties of 3D printed flexible materials showed that Polypropylene (PP) material from Ultimaker is a suitable candidate for mmWave flexible modules, with a low loss tangent of 0.001, an elastic modulus of 230 MPa, and a Poisson’s ratio of 0.40. Additionally, inkjet printed microstrip lines on 3D printed PP substrate were tested for their reliability and performance, and it was found that 6 layers of silver ink provide a good balance of electrical performance and mechanical reliability. Finally, the proposed low temp IC alignment and assembly process, along with the “smart” packaging topology used in the flexible on-package phased array with a microfluidic channel, lays the groundwork for the development of next-generation 5G mmWave broadband flexible hybrid electronics (FHE), flexible multi-chip module (MCM), and phased-array on-package modules for wearable and conformal smart skin, digital twin and massive MIMO applications.

## Data Availability

The datasets used and/or analysed during the current study available from the corresponding author on reasonable request.
